# Concurrent Presentation of Nocardia abscessus Infection and Pyoderma Gangrenosum Following Trauma

**DOI:** 10.7759/cureus.103233

**Published:** 2026-02-08

**Authors:** Igor Dumic, Ronin Joshua S Cosiquien, Joshua Jagodzinski, Danielle Alejandra Vargas Cardozo, Reginald Cosiquien, David Ladin, Andrea Boni, Libardo Rueda Prada, Milena Cardozo

**Affiliations:** 1 Hospital Medicine, Mayo Clinic Health System, Eau Claire, USA; 2 Internal Medicine, University of Minnesota School of Medicine, Minneapolis, USA; 3 Medicine, The Bolles School, Jacksonville, USA; 4 Family Medicine, Mayo Clinic Health System, Eau Claire, USA; 5 Pathology, Mayo Clinic Health System, Eau Claire, USA; 6 Hospital Medicine, Mayo Clinic Alix School of Medicine, Eau Claire, USA

**Keywords:** corticosteroid, gardening, immunosuppression, mechanical injury, nocardia abscessus, pyoderma gangrenosum, skin ulcer

## Abstract

Pyoderma gangrenosum (PG) is a rare neutrophilic dermatosis characterized by painful skin ulcers with undermined borders and surrounding erythema. Cutaneous nocardiosis can present similarly, as ulcerative lesions, often following mechanical trauma, which is a shared risk factor for both conditions. In this case report, we describe an elderly patient on low-dose corticosteroids for polymyalgia rheumatica who developed rapidly progressive skin ulcers after sustaining a mechanical injury while gardening. The patient did not respond to broad-spectrum antibiotics. Histopathological examination of a skin biopsy revealed features consistent with PG, while culture of the same biopsy grew *Nocardia abscessus*. The patient was treated with a combination of high-dose corticosteroids, trimethoprim-sulfamethoxazole, and doxycycline, leading to complete clinical recovery with residual post-inflammatory hyperpigmentation.

This case underscores mechanical trauma as a shared risk factor for the development of both PG and cutaneous nocardiosis. We hypothesize that the patient developed PG through the pathergy phenomenon following the initial injury and concomitantly developed *N. abscessus *infection. Chronic low-dose corticosteroid therapy likely was an additional risk factor for the development of cutaneous nocardiosis. However, the intriguing question remains whether *N. abscessus* infection itself could act as a trigger for the development of PG.

## Introduction

Pyoderma gangrenosum (PG) is a rare neutrophilic dermatosis characterized by painful skin ulcers with undermined borders and surrounding erythema [1]. Diagnosis typically requires a biopsy, preferably obtained from the edge of the ulcer, along with exclusion of infectious causes. Treatment focus is on pain relief, infection prevention, and the promotion of skin regrowth [2]. The first-line treatment for PG involves the use of fast-acting immunomodulating drugs such as corticosteroids or cyclosporine [1-3]. Slower-acting biologics such as tumor necrosis factor-α and interleukin-1β have also been used for treatment of resistant forms of the disease [1-3].

Nocardiosis is an infectious disease caused by a Gram-positive bacillus belonging to the genus *Nocardia spp*. These aerobic bacteria are commonly found in soil, freshwater, and saltwater environments [4]. Nocardiosis is considered an opportunistic infection, with approximately one-third of cases occurring in immunocompromised individuals, including those with HIV, organ transplant recipients, and patients undergoing chemotherapy for malignancy [5]. The lungs, brain, and skin are the most frequently affected organs. Because nocardiosis is most commonly acquired through inhalation of *Nocardia spp. *organisms from the environment, pulmonary disease is the predominant clinical presentation. Cutaneous nocardiosis is acquired through mechanical trauma and can present in four distinct clinical forms: primary cutaneous nocardiosis, lymphocutaneous nocardiosis, cutaneous manifestations of disseminated nocardial infection, and mycetoma [6].

Mechanical injury to the skin may serve as an entry point for *Nocardia spp.* and often precedes the development of cutaneous forms of nocardiosis [5,6]. Notably, mechanical trauma is also the most common precipitating factor for PG [1-2], suggesting that these two conditions share a common risk factor. By reviewing the MEDLINE/PubMed database for articles published in English, we found no reports of the concomitant PG and *Nocardia abscessus* following a single injury.

## Case presentation

We present the case of an 87-year-old White man referred for hospital admission from a dermatology clinic due to a painful, rapidly enlarging ulcerative lesion on his right forearm. The patient’s medical history included diet-controlled type 2 diabetes mellitus, hypertension, polymyalgia rheumatica on chronic low-dose prednisone, stage II chronic kidney disease, permanent atrial fibrillation, asymptomatic moderate calcific aortic stenosis, and obstructive sleep apnea. His medications included acetaminophen 1000 mg every six hours as needed for pain, apixaban 5 mg twice daily, losartan 100 mg daily, and prednisone 5 mg daily. He reported allergies to atorvastatin, rosuvastatin, and simvastatin. He quit smoking more than 30 years ago, consumed approximately four alcoholic drinks weekly, and denied illicit drug use. He had no recent travel outside Wisconsin, no pets, and no reported tick exposure.

**Figure 1:**
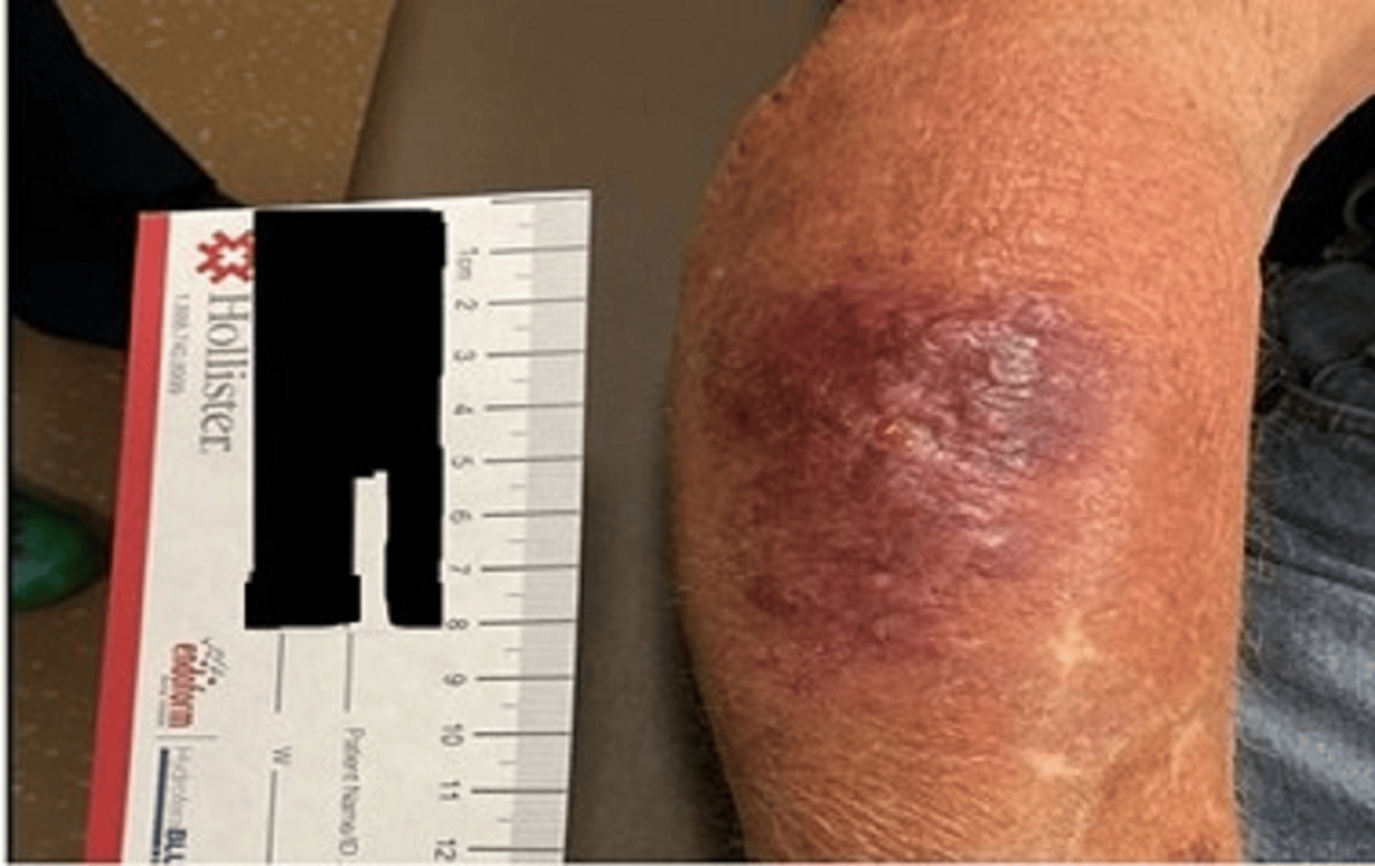
Initial evaluation of the right forearm lesion This image was obtained four days after the initial injury while gardening.

Ten days prior to admission, the patient sustained a mechanical injury in his right forearm while gardening. Over the subsequent three days, the lesion enlarged, and he developed persistent serosanguineous drainage, expanding erythema, and mild localized tenderness as illustrated in Figure 1. He denied fever, chills, or systemic symptoms. Vital signs were normal at the initial visit. The patient presented to urgent care, where examination revealed a 3 cm erythematous lesion with sanguineous drainage and no fluctuance or abscess. Motor and sensory examinations were normal, with palpable pulses. He was discharged on cefadroxil 500 mg twice daily for 10 days for presumed cellulitis.

**Figure 2:**
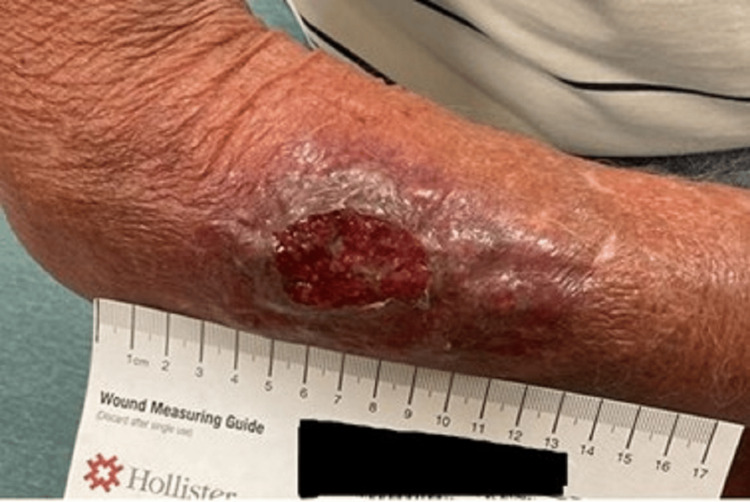
Right forearm lesion The lesion demonstrates a superficial breakout of the skin associated with a violaceous and indurated area of approximately 6 cm in diameter, with an additional 4 cm of surrounding erythema. This lesion progressed while the patient was taking cefadroxil.

Two days later (five days before admission), the patient returned to urgent care with worsening erythema, swelling, and pain. Examination showed a violaceous, indurated lesion approximately 6 cm in diameter with an additional 4 cm of surrounding erythema. A 1 mm to 2 mm central superficial opening was present, with minimal sanguineous drainage (Figure 2). 

**Figure 3:**
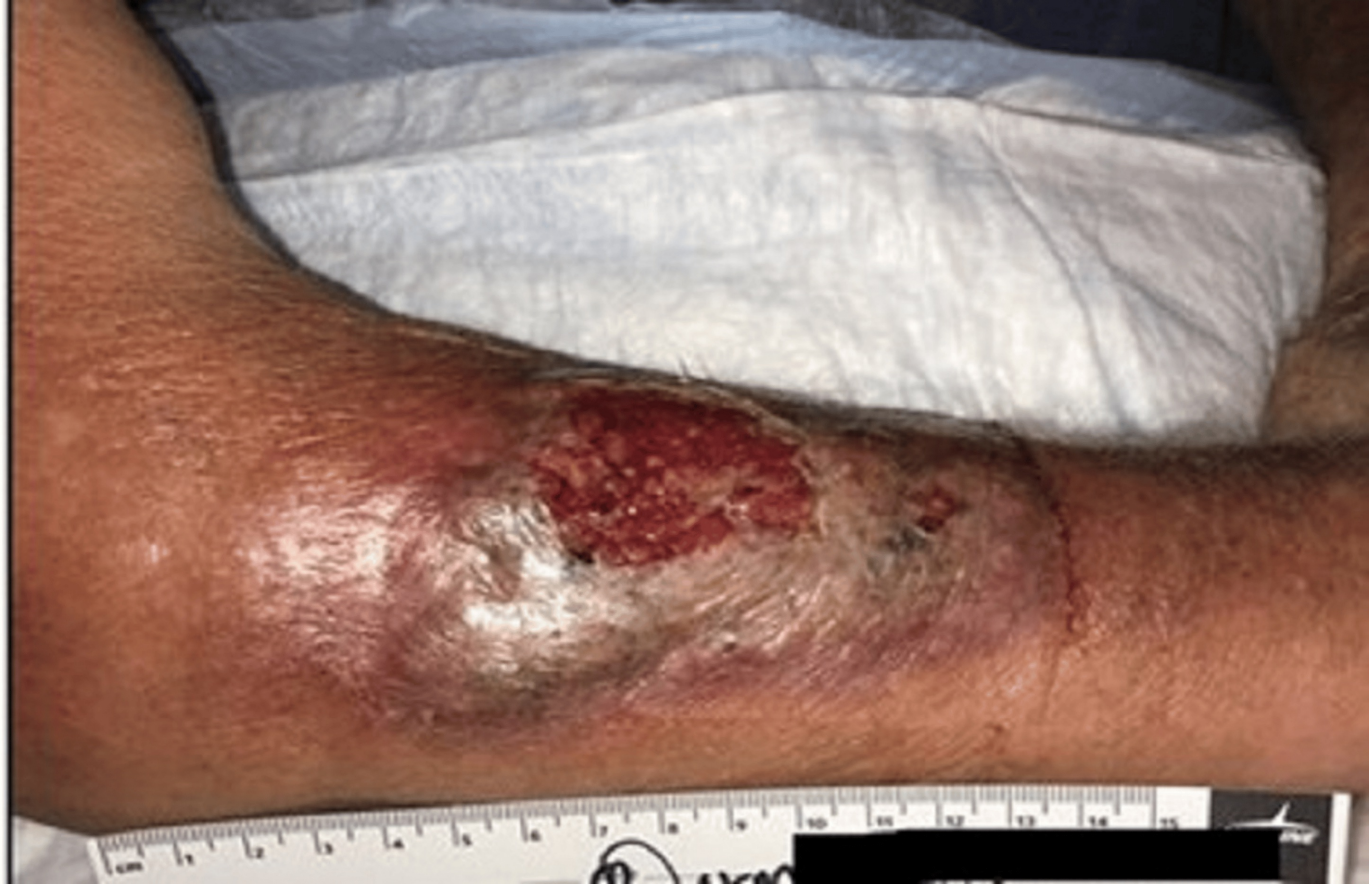
Progression of the lesion The lesion in this picture measured 12 × 6.5 cm on the right forearm and was characterized by epidermal loss with exposed granulation tissue, sanguineous drainage, and a border of violaceous erythema. The lesion was worsening despite broad-spectrum antibacterial coverage with cefadroxil and clindamycin.

Point-of-care ultrasound demonstrated cobblestoning without fluid collection. Clindamycin was added to cover for empiric methicillin-resistant Staphylococcus aureus (MRSA) coverage, and a wound culture was obtained. Despite dual antibiotic therapy, his symptoms progressed, and the lesion had expanded to 10 cm × 7 cm, with purpuric discoloration and beefy granulation tissue. The X-rays of the right forearm ruled out fracture, foreign body, or osteomyelitis. The patient then went to the dermatology clinic for evaluation, at which point the lesion measured 12 cm × 6.5 cm (Figure 3). 

**Figure 4:**
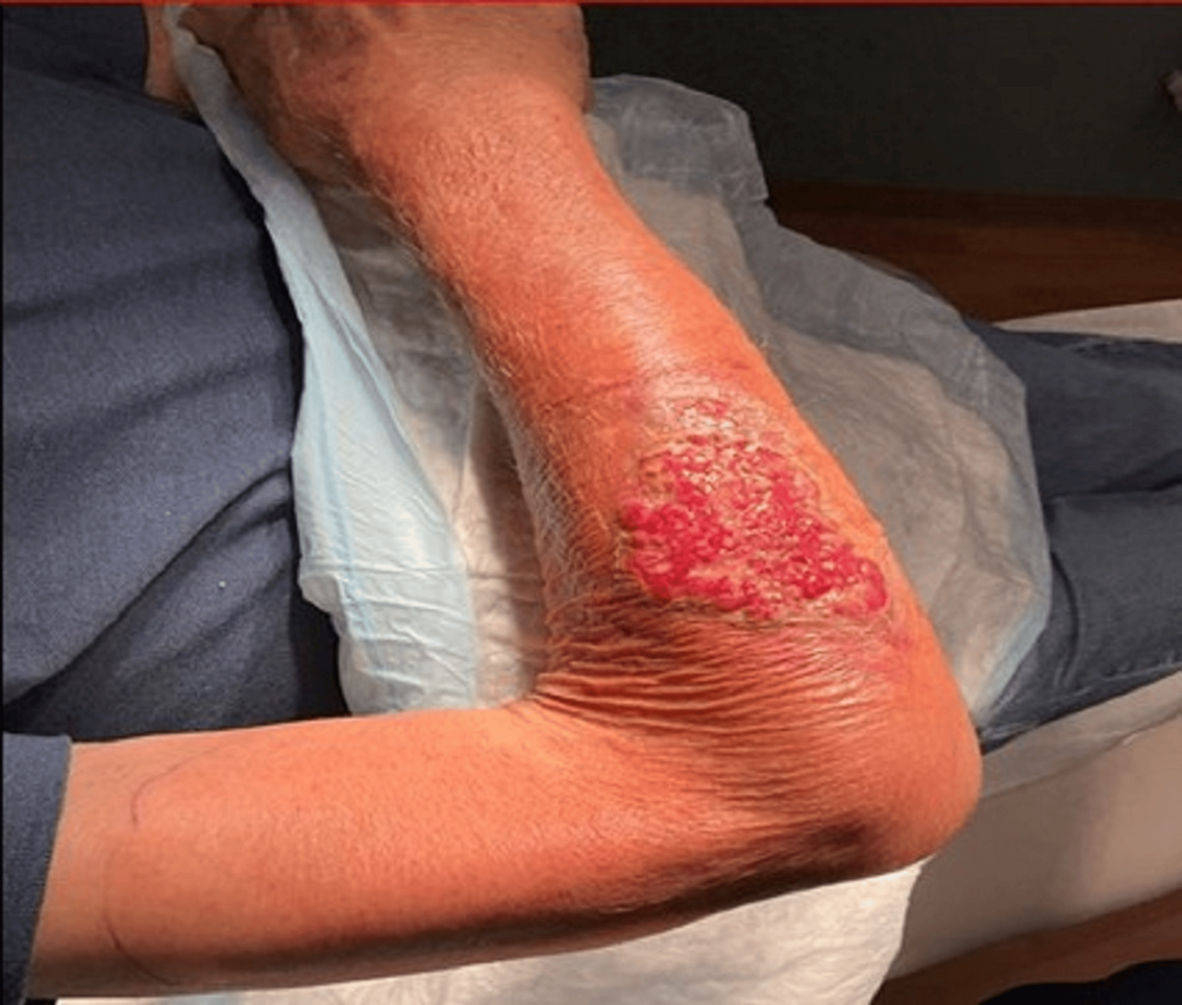
Right forearm lesion demonstrating a large ulcerated and pustular wound associated with erythema and warmth extended proximally

The epidermis had sloughed, revealing granulation tissue with sanguineous drainage and violaceous peripheral erythema. The affected area extended from the distal forearm to the upper biceps. Peripheral pulses and sensation were intact. Range of motion was preserved except for mild restrictions in the wrist and elbow. A punch biopsy was performed, and the patient was referred for hospital admission due to lesion progression and persistent pain despite antibiotics. Upon admission, the patient appeared nontoxic, alert, and hemodynamically stable. The cardiovascular exam revealed an irregularly irregular rhythm and a systolic murmur. The skin exam demonstrated a large ulcerated and pustular wound with sanguineous discharge and marked tenderness. Erythema and warmth extended proximally to the upper arm (Figure 4). 

**Figure 5:**
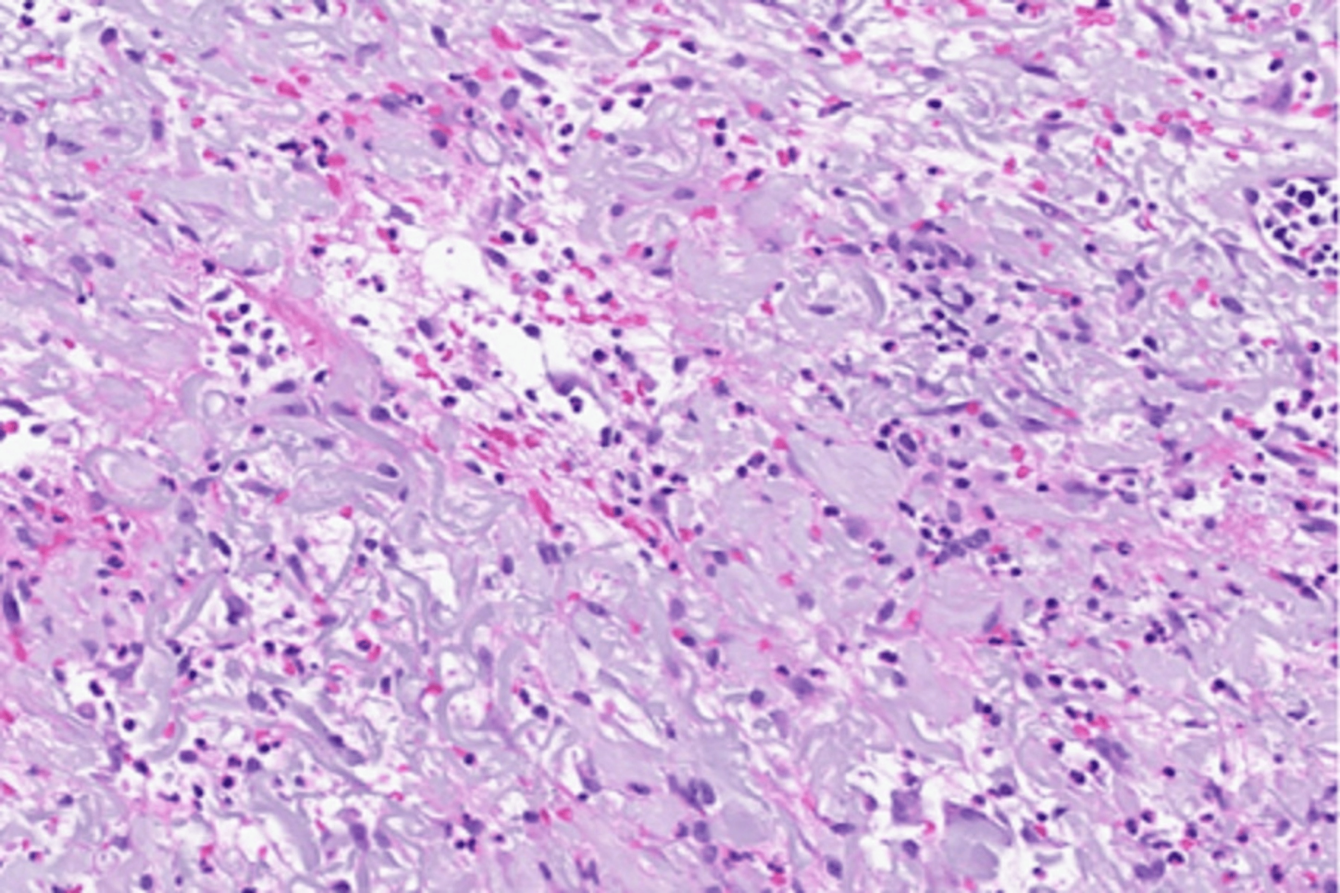
Skin biopsy The biopsy revealed superficial and deep dermal inflammation composed of lymphocytes and neutrophils with negative special stains for microorganisms (including AFB, PAS, and Gram stain). AFB: Acid-fast bacillus, PAS: Periodic acid–Schiff

General surgery was consulted, but found no evidence of necrotizing fasciitis or compartment syndrome. Given the rapid lesion progression, pain out of proportion to findings, and lack of response to antibacterial antibiotics, PG was suspected. Prednisone was increased from a baseline of 5 mg daily to 60 mg daily. Within 48 hours of the administration of steroids, the lesion began to regress, supporting a diagnosis of PG. A skin biopsy showed superficial and deep dermal inflammation composed of lymphocytes and neutrophils with negative special stains for microorganisms, including acid-fast bacillus (AFB), periodic acid-Schiff (PAS), and Gram stain (Figure 5).

**Figure 6:**
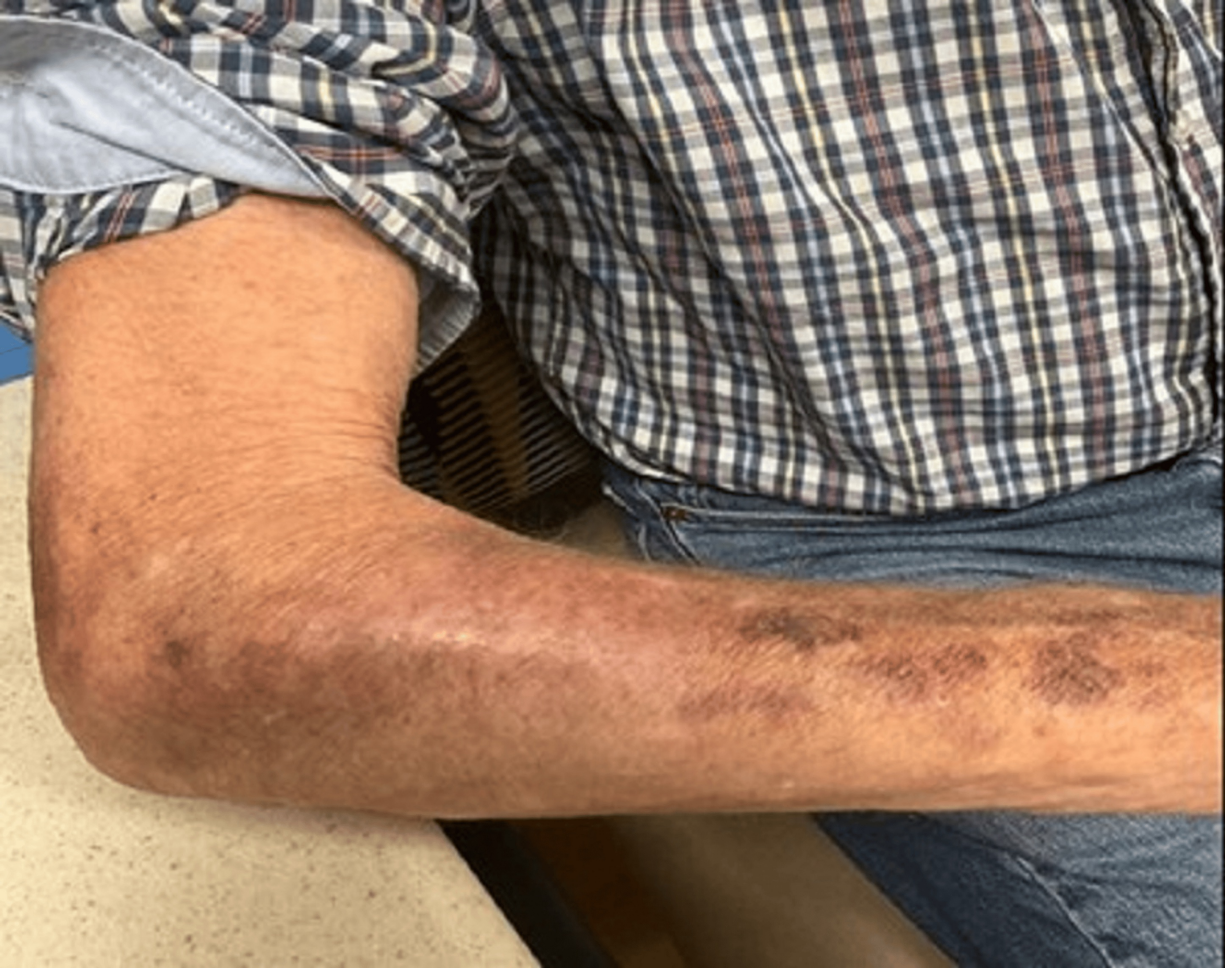
Resolution of the right forearm lesion after 10 weeks of treatment with post-treatment pigmentation

Tissue culture (tests performed at Mayo Clinic Medical Laboratories) later grew *Nocardia spp.*, at which point antimicrobial therapy was started with doxycycline 100 mg twice daily and trimethoprim-sulfamethoxazole 800/160 mg daily. Once final culture results confirmed *N. abscessus* complex with sensitivities, the patient’s therapy was continued with doxycycline for three months, and prednisone was gradually tapered down to his baseline of 5 mg daily for polymyalgia rheumatica. At follow-up two weeks before completing antibiotics (10 weeks from initial presentation), the wound showed significant clinical improvement, although with significant residual hyperpigmentation (Figure 6). 

Since PG can be associated with inflammatory bowel disease, a colonoscopy was performed, as well as fecal calprotectin, which was normal. Malignancy evaluation, including CT of the chest, abdomen, and pelvis with IV contrast, was negative for any lesions. Prostate-specific antigen was within normal range. A comprehensive autoimmune workup was negative for autoimmune diseases associated with PG. Comprehensive laboratory testing is detailed in Table 1. Given the lesion’s rapid development, significant improvement with corticosteroids, and supportive histopathology, we concluded that the primary diagnosis is PG, likely triggered by mechanical trauma and N. abscessus. Alternatively, nocardiosis might be a superinfection on the background of PG.

**Table 1:** Comprehensive workup performed for differential diagnosis of cutaneous nocardiosis and PG ANCA: Antineutrophil cytoplasmic antibody, PG: Pyoderma gangrenosum

Test	Result	Reference range
Hemoglobin	12.3 g/dL	12-14 d/dL
Platelet count	240 × 10⁹/L	150-300× 10⁹/L
Leukocytosis	13.4 × 10⁹/L	4-10 × 10⁹/L
C-reactive protein	15.4 mg/L	<5 mg/dL
Erythrocyte sedimentation rate	41 mm/h	<10 mm/h
Sodium	135 mmol/L	135-145 mmol/L
Potassium	4.3 mmol/L	3.6-5 mmol/L
Magnesium	2.0 mg/dL	1.8-2.4 md/dL
Bicarbonate	27 mmol/L	26-30mmol/L
Anion gap	10	<12
Creatinine	1.17 mg/dL	0.5-1.2mf/dL
Glomerular filtration rate (estimated)	61 mL/min/1.73 m²	>90ml/min
Aspartate aminotransferase	18 U/L	15-40 U/L
Alanine aminotransferase	11 U/L	15-40 U/L
Wound cultures/biopsy	N. abscessus	
Non-tuberculous mycobacterial cultures	Negative	
Sporothrix schenckii	Negative	
Herpes simplex virus swab	Negative	
Varicella-zoster virus swab	Negative	
Hepatitis A serology	Negative	
Hepatitis B antigen/antibody	Negative	
Hepatitis C antibody	Negative	
HIV test	Negative	
Syphilis rapid plasma reagin	Negative	
Borrelia burgdorferi PCR and antibodies	Negative	
Tularemia antibodies	Negative	
Anaplasma phagocytophilum	Negative	
Ehrlichia chaffeensis	Negative	
Antinuclear antibody screen	Negative	
Cytoplasmic antineutrophil cytoplasmic antibody (ANCA)	Negative	
Perinuclear ANCA	Negative	
Cyclic citrullinated peptide antibody	<15 U	Negative
Rheumatoid factor	<15 IU/mL	Negative
Myeloperoxidase antibody	<2 U	Negative
Proteinase 3 antibody	0.8 U	Negative
Phospholipid antibody IgG	<9.4 GPL	Negative
Phospholipid antibody IgM	<9.4 MPL	Negative
Prostate-specific antigen	Normal	
Carcinoembryonic antigen	Normal	
Cancer antigen 19-9	Normal	
Serum protein electrophoresis	Normal	
Urine protein electrophoresis	Normal	
CT chest/abdomen/pelvis with IV contrast	Negative for malignancy	
Brain MRI	Negative for malignancy	

## Discussion

Our case is compelling and, to the best of our knowledge, represents the only reported instance of overlapping cutaneous *N. abscessus *infection and PG Cutaneous nocardiosis is typically acquired through direct inoculation via exposure to contaminated soil, while PG is frequently triggered by mechanical trauma, both of which were present in our patient. This overlap underscores the need to consider both infectious and autoinflammatory processes in complex cutaneous lesions, particularly in individuals with relevant risk exposures.

In addition to mechanical trauma, PG may be precipitated by infection; however, infection is not the underlying cause of the disease. Importantly, infectious etiologies must typically be excluded prior to establishing a diagnosis of PG. The condition is primarily driven by autoinflammatory and dysregulated immune mechanisms and aberrant neutrophil function [7]. A key feature of PG is pathergy, a phenomenon in which minor trauma, including infections or surgical procedures, triggers the development of characteristic ulcers. In this context, soft tissue infection may serve as a nonspecific trigger for PG in susceptible individuals. However, the resulting ulcers are not infectious in origin [7-9]. Differentiating PG triggered by infection from ulcers caused by primary infectious agents is critical, as the therapeutic approaches diverge significantly. Immunosuppressive therapy, the cornerstone of PG management, can exacerbate true infections and lead to adverse outcomes if the diagnosis is incorrect. As illustrated in our case, mechanical injury or infection with* N. abscessus* may have acted as a triggering event for the development of PG. In such scenarios, patients may require concurrent management with immunosuppressive agents to treat PG and appropriate antimicrobial therapy to address secondary bacterial superinfection of the lesion.

Given our patient’s possible immunosuppression from steroid therapy, environmental exposure to soil during gardening, and clinical presentation, multiple diagnoses were considered. Initially, given the presentation of a lesion with erythema, drainage, and tenderness with a history of diabetes and chronic steroid use, a primary skin and soft tissue infection appeared most likely. This diagnosis became less compelling as the patient had failed treatment with multiple trials of broad-spectrum antibiotics.

The minimal surrounding erythema and no streaking or pain in his fingers and hands, with otherwise normal function of the affected extremity, ruled out necrotizing fasciitis and compartment syndrome. Imaging of the right lower extremity ruled out osteomyelitis or foreign body. Calciphylaxis was a consideration; however, this was less likely given mild renal impairment and the absence of end-stage renal disease. This was further ruled out as histopathology did not reveal calcifications, thrombosis, or other findings that are consistent with calciphylaxis [10].

A comprehensive autoimmune workup was pursued in search of a cutaneous manifestation of autoimmune disease, as our patient had a history of polymyalgia rheumatica, but it was unrevealing. Colonoscopy was negative for inflammatory bowel disease as well as fecal calprotectin. A CT scan of the chest, abdomen, and pelvis with the use of IV contrast ruled out intrathoracic or intra-abdominal malignancy. Cutaneous tularemia was a consideration due to the possibility of acquiring it through tick bites in endemic areas; however, the workup was negative. Finally, the cultures of the skin biopsy led to the diagnosis of cutaneous nocardiosis and PG.

In this case, the patient had an additional risk factor for nocardiosis in the form of chronic prednisone therapy for polymyalgia rheumatica. Chronic corticosteroid therapy is a well-established risk factor for *Nocardia* infection. Substantial evidence from retrospective cohort and case-control studies demonstrates a strong association between systemic corticosteroid use and nocardiosis, particularly among patients treated for autoimmune, inflammatory, or neoplastic conditions [11,12]. The increased susceptibility to nocardiosis in patients receiving long-term corticosteroids is primarily attributed to corticosteroid-induced impairment of cell-mediated immunity. This includes functional suppression of macrophages and T lymphocytes, which play a central role in host defense against intracellular pathogens such as *Nocardia spp.* Corticosteroids downregulate the expression of proinflammatory cytokines and chemokines, inhibit antigen presentation, and attenuate the activation and effector functions of both macrophages and T cells [13]. In addition, chronic corticosteroid use can lead to CD4+ lymphopenia and reduced serum IgG levels, further compromising the immune system’s ability to control opportunistic infections [12,13].

Nocardiosis is a difficult infection to treat, with no established guidelines of treatment given its variable susceptibility patterns [14]. Skin disease and non-complicated pulmonary infection are treated with single antibiotic therapy, while more severe disease requires a multidrug therapy regimen. Sulfonamides such as trimethoprim-sulfamethoxazole (TMP-SMX) have been the cornerstone of therapy. Oxazolidinones such as linezolid or tedizolid are alternatives; however, myelosuppression may limit their prolonged use. Additional agents such as minocycline, fluoroquinolones, and amoxicillin-clavulanate are oral options that have shown activity against *Nocardia spp.* in vitro [1-6]. These could be considered as alternatives to TMP-SMX only when the initial clinical response is favorable. Multidrug regimens include TMP-SMX and the addition of third-generation cephalosporins (ceftriaxone, cefotaxime), imipenem, or amikacin [1-6]. Antibiotic therapy is adjusted based on antimicrobial susceptibility results. Length of treatment is usually two to six months for most cases, but extended regimens are required in disseminated disease and those with central nervous system involvement.

## Conclusions

This case report highlights the coexistence of two rare clinical entities, both of which share mechanical trauma as a common risk factor. Additionally, environmental soil exposure represents a unique risk factor for nocardiosis. Clinicians should recognize that PG is often triggered by mechanical trauma through the pathergy phenomenon. As illustrated in this case, nocardiosis can develop concurrently at the site of injury. Alternatively, nocardiosis may have triggered PG, or, if not the primary cause, it could have exacerbated the lesion, making it more severe.

In clinical scenarios such as the one described here, a combination of immunosuppressive and antimicrobial therapy may be necessary to achieve favorable outcomes. Careful vigilance is essential when managing patients with ulcerative skin lesions, as immunosuppressive treatment for inflammatory conditions like PG can worsen underlying infections. Further studies are warranted to explore whether infections such as nocardiosis may serve as potential triggers for the development of PG.
